# Masswe Colonic Haemorrhage from a Solitary Caecal Varix

**DOI:** 10.1155/1996/65719

**Published:** 1996

**Authors:** K. R. L. Shaper, M. Jarmulowicz, R. Dick, N. D. Cuthbert, B. R. Davidson

**Affiliations:** 1 Department of Surgery Royal Free Hospital Pond Street, Hampstead London NW3 2QG United Kingdom; 2 Departments of Histopathology Royal Free Hospital Pond Street, Hampstead London NW3 2QG United Kingdom; 3 Departments of Radiology Royal Free Hospital Pond Street, Hampstead London NW3 2QG United Kingdom

## Abstract

A 51 year old lady with chronic active hepatitis presented with massive lower gastrointestinal tract bleeding. Angiography demonstrated a solitary varix in the caecum which was found at laparotomy to be entering the bowel wall at the site ofadhesions from a previous appendicectomy. The portal pressure was found to be raised. Aright hemicolectomy stopped the blood loss, but she subsequently died ofliver failure. Solitary colonic varices associated with adhesions are extremely rare and their optimal management has not been established.


*HPB Surgery*, 1996, Vol.9, pp.253–256
Reprints available directly from the publisher
Photocopying permitted by license only

© 1996 OPA (Overseas Publishers Association)
Amsterdam B.V. Published in The Netherlands
by Harwood Academic Publishers GMbH
Printed in Malaysia

### CASE REPORT

### Massive Colonic Haemorrhage from a Solitary Caecal Varix

K. R. L. SHAPER, M. JARMULOWICZ*, R. DICK+, N. D. CUTHBERT and B. R. DAVIDSON

Departments of Surgery, *Histopathology and +Radiology, Royal Free Hospital, Pond Street, Hampstead, London NW3 2QG

*(Received 20 January 1994)*

A 51 year old lady with chronic active hepatitis presented with massive lower gastrointestinal tract bleeding. Angiography demonstrated a solitary varix in the caecum which was found at laparotomy to be entering the bowel wall at the site of adhesions from a previous appendicectomy. The portal pressure was found to be raised. A right hemicolectomy stopped the blood loss, but she subsequently died of liver failure. Solitary colonic varices associated with adhesions are extremely rare and their optimal management has not been established.

KEY WORDS: Colonic bleeding varices

### INTRODUCTION

Bleeding from colonic varices is rare, but is recognised as a complication of portal hypertension. A recent review documented 127 cases in the literature[1]. Colonic varices are usually multiple and may occur through adhesions at the site of previous surgery[2]. We report a unique case of massive colonic bleeding from a solitary varix at the site of an appendicectomy.

### CASE REPORT

A 51 year old lady was admitted to hospital with profuse fresh rectal bleeding. She had a history of chronic active hepatitis diagnosed in 1973 on liver biopsy and was maintained on Prednisolone 5mg and Spironolactone 100mg daily. Her past medical history included an appendicectomy at the age of fourteen.

On admission she was shocked, with pulse of 130 per min and an unrecordable blood pressure.

*Correspondence to*: B.R. Davidson, Department of Surgery, Royal Free Hospital, Pond Street, London. NW3 2QG.

Blood tests revealed Hb 8.6 g/dl(11.5–15.5), WCC 12.8(4.5–11), Platelets 111(140–500), Urea 5.7mmol/l (3–6.5), Na 153mmol/l (135–145), K 5.7mmol/l (3.5–5.0), Bilirubin 12.0 mcmol/l(5–17), Albumen 24g/l(35–50), ALP69u/l(35–130), AST 41u/l (5–40), ALT 35u/l(5–40), PT 41 sec (12–16), PTTK 154 sec (30–40), INR 4.8, FDP<0.5 (2–4).

Active resuscitation was commenced and a gastroscopy was performed. No blood was seen in the stomach or duodenum and there was no evidence of oesophageal varices. Mesenteric angiography was then undertaken to delineate the source of bleeding. This revealed a large, anomalous vein running from the region of the caecum, laterally in the right paracolic gutter, up to the inferior vena cava [Figs. 1&2]. The portal vein was patent and there were no varices at other sites. The spleen was of normal size. A technetium labelled red cell scan confirmed that the bleeding was from the right side of the abdomen. Despite rapid transfusion and clotting factor replacement, the bleeding continued and a laparotomy was performed. At operation the liver was noted to be cirrhotic. The colon was distended with blood which was also present in the terminal ileum. Adhesions were found between the abdominal wall and caecum at the site of her previous appendicectomy and running

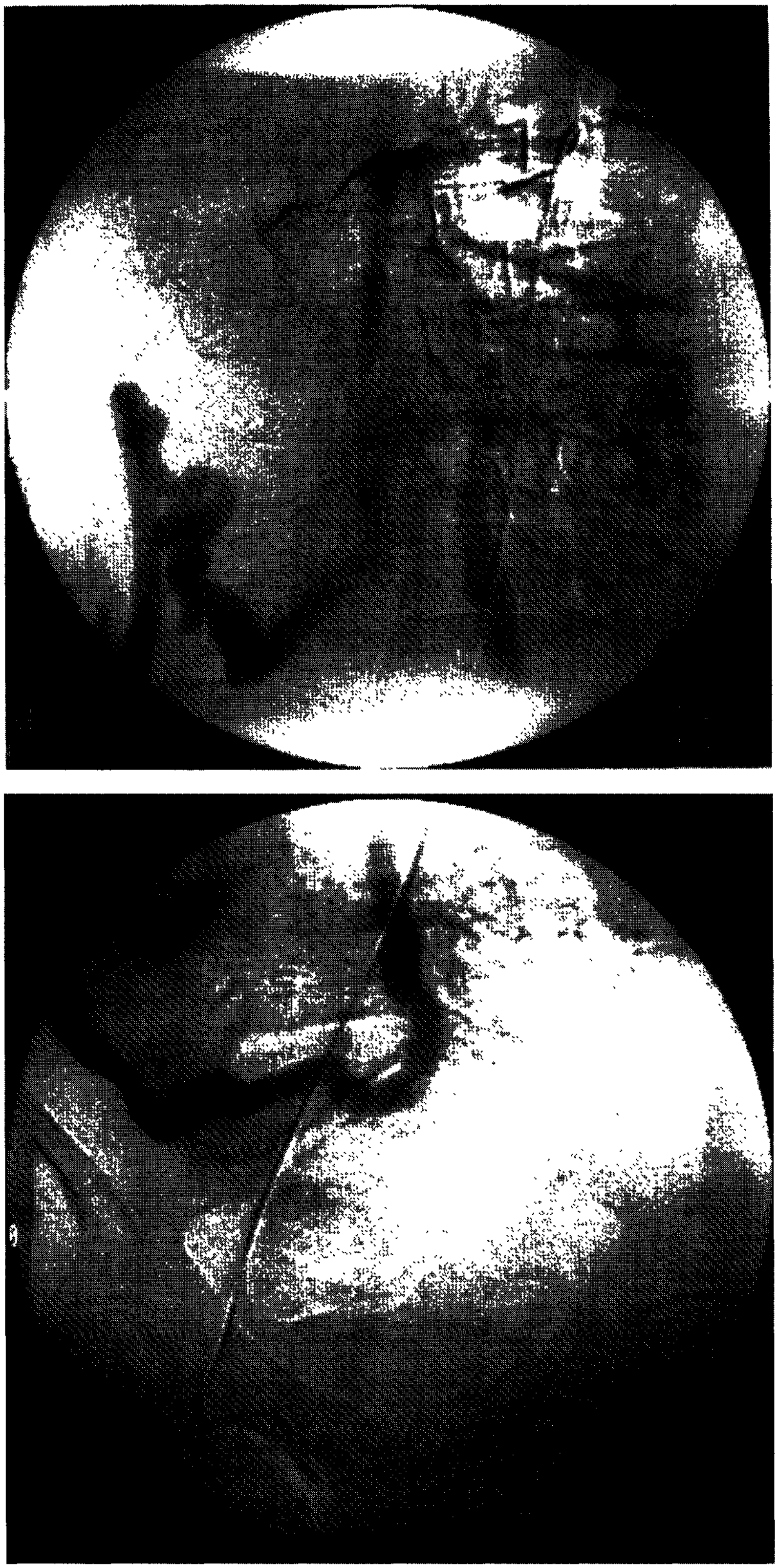

**Figures 1 and 2** Venous phase of the mesenteric angiogram, showing the varix in the right side of the abdomen and pelvis.

through these, into the caecal wall was a 2 cm diameter varix. The portal vein pressure was 24 mmHg. A right hemicolectomy was performed.

Examination of the specimen, which consisted of 22 cms of terminal ileum and 13 cms of colon, revealed a 1cm diameter tortuous vessel running alongside the colon within the pericolic fat. At the caecum it passed through the muscularis propria to form a 2 cm diameter varix within the submucosa, opposite the ileo-caecal valve. The overlying mucosa was intensely congested and nodular, but no definite ulceration or perforation into the varix could be identified macroscopically.

Microscopically the varix directly abutted the muscularis propria. Its wall was arterialized with the formation of irregular elastic laminae and smooth muscle bundles. The mucosa overlying the varix showed early necrosis (Fig.3). There was marked congestion and dilatation of submucosal veins in the adjacent small and large bowel. Bleeding was assumed to have arisen from the congested and necrotic mucosa.

Postoperatively she was transferred to ITU, and had no further GI blood loss. She developed acute renal failure, requiring haemofiltration and progressive liver failure. At nine days postoperatively she developed a series of cardiac tachyarrhythmias that were unresponsive to amiodarone and cardioversion. She became clinically septic and died 10 days post-operatively of multi-organ failure.

Postmortem examination revealed acute tubular necrosis, multiple infarctions in a cirrhotic liver, and a subendocardial infarct. It was considered that these had all resulted from the profound hypotension at the time of her massive GI bleed. There were no varices at other sites and no macroscopic evidence of a septic focus.

### DISCUSSION

This is the second well documented case of massive GI bleeding originating from a solitary caecal varix[3]. In this case the varix was associated with adhesions from a previous appendicectomy. This case differs from previous reports on variceal haemorrhage from post appendicectomy adhesions in that the varix was solitary, previous reports demonstrating multiple varices communicating between portal and systemic circulation, enlarged by the presence of portal hypertension[4&5].

Diagnosis of the cause of colonic bleeding can be difficult. In this case the abnormal vein was seen on angiography, but no intra luminal contrast was apparent. This problem has been described before[2] and is

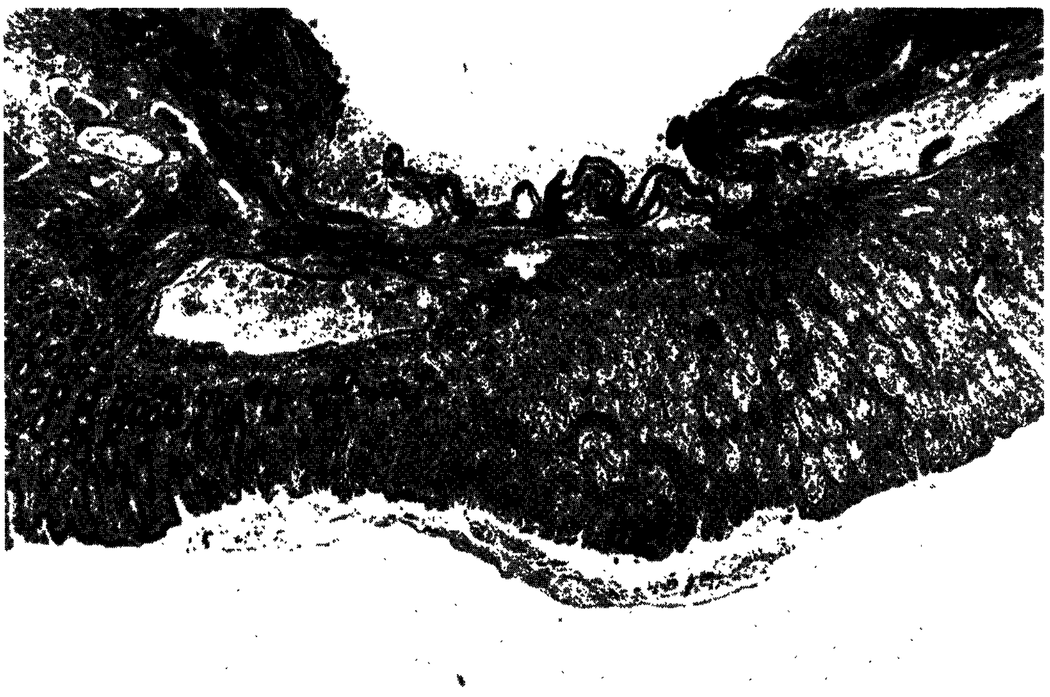

**Figure 3** Caecal varix abutting muscularis mucosae with overlying early mucosal necrosis. (HEVG × 100)

probably due to the dilution of contrast which occurs when imaging the venous phase of an arterial injection of contrast and the intermittent nature of the bleeding. The site of colonic bleeding may therefore be better assessed by labelled red cell scanning but neither investigation is useful unless the patient is actively bleeding[6]. Management of patients with liver disease and massive GI haemorrhage is associated with a very high mortality, mainly due to liver failure and sepsis. The absence of oesophageal varices and the normal sized spleen on angiography did not suggest the presence of portal hypertension pre-operatively, although this was confirmed by portal pressure measurement at operation. The operative management for colonic varices falls into two groups–colonic resection and portosystemic shunting. These therapeutic modalities have not been directly compared, but an analogous situation which has been studied is that of bleeding from oesophageal varices in which oesophageal transection has been shown to have a lower mortality and encephaopathly rate than portosystemic shunting[7]. However, with colonic variceal bleeding, bowel resection has the advantage over shunting that it is an operation done by the majority of general surgeons.

If portal hypertension can be confirmed pre-operatively then another treatment option is transjugular intrahepatic portosystemic shunt (TIPS). This has made an impact in the acute management of bleeding oesophageal and gastric varices secondary to portal hypertension but has not yet been applied to colonic variceal bleeding.

Liver transplantation was also considered with the development of liver failure. Although suspected sepsis precluded transplantation in this case, it would be a treatment option after initial control of the colonic bleeding by TIPS and this combination may be the ideal therapeutic option in patients with advanced liver disease and massive colonic haemorrhage[8].

### REFERENCES

1. Orozco H, Takahashi T, Mecando M, Prado-Orozco E, Ferral H, Hernandez-Ortiz J, Esquivel E. (1992) Colorectal variceal bleeding. *J. Clin. Gastrol* **14** (2) 139–143.
2. Monare A.C, Waltman A.L, Vandersalm T.J, Linton R.R, Levine F.H, Abbott W.M. (1976) Gastrointestinal haemorrhage from adhesion related mesenteric varices. *Ann Surg* **183**: 24–29.
3. Patel K.R, Wu T.K, Powers S.R. (1979) Varices of the colon as a cause of Gastrointestinal haemorrhage. Dis Colon Rectum, **22**: 321–323.
4. Bloor K, Orr W. (1961) A case of haemorrhage from varices in the small intestine due to portal hypertension. *Br J Surg*, **48**: 423.
5. Manzi D, Samanta A. (1985) Adhesion related colonic varices. *J Clin Gastroenterol*. **7**: 71–75.
6. Gudjonsson H, Zeder D, Gameli R.L, Kage M.D. (1986) Colonic varices. Report of an unusual case diagnosed by radionuclide scanning with a review of the literature. *Gastroenterology*. **91**: 1543–1547.
7. Osborne D.R, Hobbs K.E.F. (1981) The acute treatment of haemorrhage from oesophageal varices: a comparison of oesophageal transection and staple gun anastomosis with mesocaval shunt. *Br J Surg*. **68**: 734–737.
8. Ring E.J, Lake J.R, Roberts J.P, Gordon R.L, LaBerge J.M, Read A.E, Sterneck M.R, Ascher N.L. (1992) Using transjugular intrahepatic portosystemic shunts to control variceal bleeding before liver transplantation. *Ann Int Medicine* **116**: (4) 304–309.

